# Hepatitis A Virus Seropositivity in Nurses and Paramedical Personnel at a University Hospital in North Iran

**DOI:** 10.5812/ircmj.10157

**Published:** 2013-05-05

**Authors:** Masomeh Bayani, Mahmoud Sadeghi, Narges Kalantari, Abdolah Sayadmanesh

**Affiliations:** 1 Infectious Diseases Research Center, Babol Medical University, Babol, IR Iran; 2 Cellular and Molecular Biology Research Center, Babol Medical University, Babol, IR Iran; 3 Infectious Diseases Department, Faculty of Medicine, Babol Medical University, Babol, IR Iran

**Keywords:** Hepatitis A virus, Nurses, Allied Health Personnel, HIV Seroprevalence

## Abstract

**Background:**

The status of hepatitis A virus (HAV) among health care workers has not been studied yet in Iran.

**Objectives:**

This study aimed to evaluate the HAV seropositivity among the healthcare personnel in Ayatollah Rohani Hospital, Babol, Iran, according to age, number of working years, and other demographic data.

**Patients and Methods:**

This cross sectional study was performed on all nurses, nurses' aid, and paramedical technicians at Ayatollah Rohani Hospital, Babol, from March 2011 to March 2012. Blood was obtained from all cases (466) and the sera were separated. All serum samples were tested for anti-HAV antibodies (IgG) by enzyme-linked immunosorbent assay (ELISA). Data were analyzed by logistic regression analysis using SPSS software, version 18.

**Results:**

330 out of 466 (71%) persons were positive for anti-HAV antibodies (IgG) with no significant difference between females (71.5%) and males (70%) (P = 0.89, 95% CI. 0.533-2.083). The lowest sero-prevalence rate was observed in the 20-29 year age group (57.8%). Seropositivity for HAV significantly increased with age (P < 0.000, 95% CI. 1.626-3.262), 77.4% for 30-39 years and 85.3% for more than 40 years. The seropositivity rate also increased significantly in accordance with the number of working years (P = 0.012, 95%CI. 1.098-2.170). 110 out of 353 (31.2%) cases were seronegative among those with less than 5 years of working time. An obvious decrease of seronegative rate of HAV was seen in those with 5-10 years (27%) and more than 10 years (14.3%) of working time.

**Conclusions:**

These findings indicate relatively high prevalence rate of HA infection among nurses and paramedical personnel at this hospital. Nevertheless, 30% of the health workers have been seronegative and are still at risk of HA infection development. Considering that the disease has more severe course as age increases, improvement of standard hygiene and prevention strategies are recommended. Furthermore, vaccination may play a significant role in the occupational health policy to protect the susceptible health care workers population in the future.

## 1. Background

Hepatitis A virus (HAV) is an enteric transmitted infection and causes acute and self-limited viral hepatitis in the world. About 1.5 million clinical cases of Hepatitis A occur per year, but the infection rate is probably higher (1). Epidemiological studies reveal that distribution and the rate of HAV infection is related to hygienic and sanitary conditions. Clinical presentation of HAV infection is highly age dependent. Although mortality rate is relatively low in children but it has a considerable fatality rate among adult population, leading to severe disease with possible hospitalization, long absence from work for several weeks, and acute fulminant HA infection development. The increasing severity of the HAV infection by age is a major problem. (1, 2). In a study from Canada, 54% of HAV hospitalizations occurred in 20–39 year-olds adults, and the overall case fatality ratio among hospitalized patients was 1.4%, which ranged from 0.4% in those < 40 years old to 12.5% in those ≥ 60 years (3). On the other hand, during the last few decades, a substantial improvement in the standard of living and general health even in rural areas has been achieved in some developing countries. Improvement of hygienic and sanitary status changed the rate of HAV infection (4-6). Several studies indicated that sero-prevalence rate of HAV infection declined in many hyper-endemic countries within the past two decades where their economic status improved. This transition to lower rates of infection is also observed in Iran which resulted significantly to a reduction of HAV infection (7, 8). For instance, a study which was performed in 1-30 years old population in a less developed district of Mazandaran province, north of Iran, showed that the overall seropositivity rate of the infection was 19.2%. In the latter study, 31.6% of young adult (18-30 years) were susceptible to the HAV infection (4). By increasing the age of general population, the susceptibility risk increased. Based on available knowledge, nosocomial transmissions of HA to healthcare workers are low. It is a rare mode because most patients with HA are hospitalized after onset of jaundice; however transmission of infection is not uncommon (9). The main source of transmission of infection to health care workers is undiagnosed HA patients hospitalized for evaluation of fever. In this situation, modes of nosocomial HAV transmission is in association with fecal incontinence of the patients, invasive procedures, food borne infection and infusion of HAV-contaminated blood products (9, 10). Furthermore, sero-prevalence rates of HAV infection among healthcare workers varied from 4% to 90% according to countries and occupations. In Iran, the first sero-prevalence study on HAV infection among healthcare workers was performed in North Iran, 2003. This study indicated that the sero-positivitiy rate of HAV infection was 90.36% (11). But according to more recent population based studies, HAV epidemiology was changed and prevalence patterns shifted to lower rates of endemicity in Mazandaran province and all-over the country (4, 7, 8). Thus, it seems that preventative strategies and other public health priorities should be reevaluated. At present, hepatitis A vaccination is not available in Iran and therefore periodic sero-prevalence studies in different populations such as healthcare workers should be performed.

## 2. Objectives

The present study was conducted to identify the prevalence rate of HA seropositivity among healthcare workers of a big teaching hospital in Mazandaran province.

## 3. Patients and Methods

### 3.1. Population Study and Sample Collection

Ayatollah Rohani Hospital is a tertiary hospital, university-affiliated medical center with 375-bed located in Babol, Northern Iran. In this study 466 individuals participated in this study, who were working as nurses, nurses' aid and paramedical personnel (laboratory technicians and other technicians) in the hospital.. This cross-sectional study was carried out from March,21, 2011 to March,20, 2012. Data on demographic (age, gender, educational level, and current residence) and occupational characteristics were collected by filling in a questionnaire interview. 

From each participant5 milliliters of venous blood was collected and transported to the designated lab for further processing. After clotting and centrifugation, the serum samples were separated and stored at -20˚C until needed. The serum samples were tested for the presence of anti-HAV total (IgG) antibodies at the clinical laboratory for serologic diagnosis, Ayatollah Rohani Hospital. The sera were tested using an enzyme-linked immunosorbent assay (ELISA) (Dia pro kit; HAV Ab, Italy). Cutoff values were obtained according to the manufacturer's instructions by reference to control sera. The sensitivity of the assay is 100% and the specificity is higher than 98%. The results were reported as positive or negative. The technicians who carried out the tests had no access to the questionnaires’ information.

### 3.2. Data Analysis and Ethical Considerations

The data were analyzed by SPSS version18. Logistic regression test was used to analyze the data and a 5% statistical significance level and 95% confidence interval (CI) were calculated and presented for variables associated with risk of infections. The sero-prevalence of HAV was determined and compared in association with risk factors including age, number of working years, different wards, residency, and educational level among the healthcare workers. In statistical analysis, cases were classified according to age, sex, duration of employment, residency, different wards, and educational status. This study was approved by letter No.839 of the Ethical Committee of the Research Council of Babol University of Medical Sciences, Babol, Iran. Written informed consent was obtained from all participants after the purpose of the study was explained in detail.

## 4. Results

**Figure 1.**
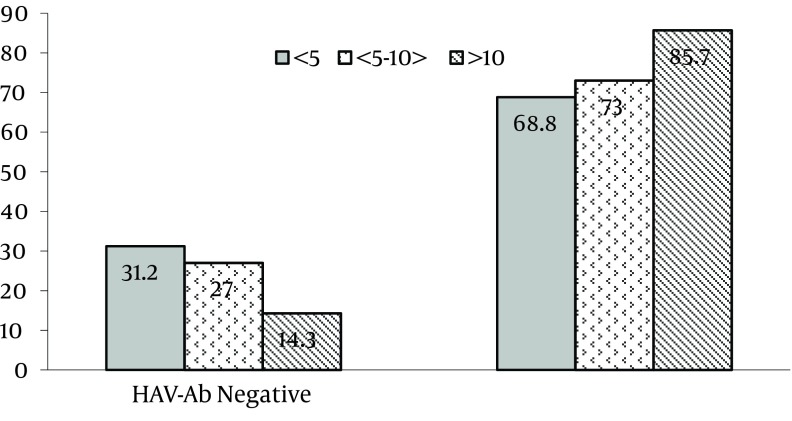
Seropositivity Rate of Hepatitis A Among Healthcare Workers Associated with their Record of Service, Ayatollah Rohani Hospital, Babol, Iran, 2011. (P = 0.012, 95%CI., 1.098-2.170)

The study had 44 out of 466 (9.4%) cases that were male and 418 (89.7%) that were females. The gender of four participants was not recorded (0.8%). The mean age of the studied population was 32.5 ± 5.52 years (ranging from 21 to 54 years). Of 466 serum samples, 330 (71%) were positive for anti-HAV IgG antibodies. The seropositivity rate was 57.8% (93 out of 161), 77.1% (192 out of 299), and 86.3% (44out of 51) among 20-29, 30-39, and < 40 year age groups, respectively, with significant differences (P < 0.000) (Table 1). There was a positive and significant relationship between seropositivity rate and aging. Furthermore, there was no association between anti-HAV seropositivity and gender, ward of employment, residency, and education level (Table 1). Moreover, the sero-prevalence rate among the studied population in association with the number of working years is shown in Figure 1. Anti-HAV antibodies were detected in 68.8% of cases who had less than 5 years of working time. The seropositivity rate was noticeably increased in individuals who had 5 to 10 and more than 10 years of working time (P = 0.012,95%CI., 1.098-2.170). On multivariate logistic regression analysis only age significantly associated with HAV seropositivity (P = 0.000, 95% CI. 1.672-3.430). Gender, residency, different wards, and level of education showed no significant contribution to the logistic model.

**Table 1.** Baseline Characteristics andSero-prevalence Rate of Anti-Hepatitis A Virus (IgG) Antibodies Among Healthcare Workers in Ayatollah Rohani Hospital, Babol, Iran

Variable	No. (%)	HAV Seropositivity, No. (%)	Odds Ratio	95% CI	P value
**Gender**					
Female	418 (89.7)	299 (71.5)	1	0.533-2.083	0.89
Male	44 (9.4)	31 (70)	0.842		
**Age Group**					
20-29	161 (34.8)	93 (57.8)	0.238	1.626-3.262	0.000
30-39	249 (53.8)	192 (77.1)	0.619		
> 40	51 (11)	44 (86.3)	1		
**Kinds of wards**					
Risk wards	55(11.8)	27 (56)	0.159	0.330-1.525	0.38
Other wards	411(88.2)	303(77.4)	1		
**Educational**					
Diploma	41 (8)	29 (70.1)	3.459	0.862-1.614	0.3
Associate degree	84 (18)	56 (66)	0.809		
B.Sc.	340 (73)	248(72.9)	1		
**Residency**					
Urban	344 (74.5)	241 (70.1)	1.336	0.813-2.116	0.27
Rural	118 (25.5)	89 (75.4)	1		

## 5. Discussion

In the current study sero-prevalence rate of HA among the healthcare workers of a hospital were determined to estimate the level of susceptibility to the infection and to identify those groups of workers at increased risk of the infection. The results showed that approximately one-third of the hospital employee were seronegative to HAV and hence susceptible to infection on exposure. The rate of susceptibility was higher (42.2%) in cases aged from 20 to 29 year group in comparison with other aged groups. The detection of frequent HAV seronegativity among healthcare workers is important due to exposure to HAV at older age, when the infection is more complicated and acute fulminant hepatitis may occur (1 - 3). Moreover, these findings also demonstrated that the sero-prevalence rate of HAV among the health care workers was relatively high (71%), but it was lower in comparison with results obtained from another study which was performed on healthcare workers in Iran (11). These differences could be explained by improvement in the standard of living and general health which led to a reduction in the rate of HAV infection among a general population at a lower age. In fact, an epidemiological shifting in Iran, particularly in Mazandaran province where this study was performed, is observed. The first epidemiological report of HAV infection in Mazandaran province was in 1997 and it was carried out among 1-15 year-old children. This study demonstrated a high prevalence rate (87 %) of HAV infection in that region (12). It also revealed that the sero-prevalence rate was 74.7 %, 86.7 %, and 90.6% in age groups 1-5, 5-10, and 12-15, respectively. Other studies which were performed in some special high risk groups had similar results to the older children and adults in the studied region (11 - 13). Also, results of a recent age-specific sero-epidemiological study among the 1-30 year-old population reported a lower infection rate especially among children, from 5.7% in age group 1-2.9 year to 34.8% in adolescents, and to 68.4 % in young adults (4). These results observed a declining rate of HAV infection as well as a transition to a less prevalent pattern of HAV infection in the Mazandarn province. The finding of our study agrees with reports published in earlier studies (4 , 14). Moreover, the seropositivity rate of HAV significantly increased by age and the highest sero-prevalence rate was observed in cases aged more than 40 years old. This result is in agreement with findings obtained from recent studies (10 , 15 , 16). On the other hand, a direct association between HAV infection and the number of working years was seen by the current study (Figure 1). It demonstrated that the HAV seronegativity rate was relatively high (31.2%) in healthcare workers who had less than 5 years of working time. Generally, personnel at this group are in 20 to 29 years of age and susceptible to acquired infection from their work place in addition to their community. These findings supported that sero-prevalence rate of HAV have declined and the changing of the epidemiology of hepatitis A virus occurred in Iran as well as in many other world regions. Additionally, the sero-prevalence of HAV was not significantly different in females (71.5%) and males (70%). These results are in agreement with some studies (17 , 18) but are in contrast with others indicating that sero-prevalence rate is higher in male (15). Also, this study did not find statistic differences between the HAV seropositivity rate in cases working at infectious disease or gastrointestinal wards with other wards. In addition, no statistically significant differences were seen between the sero-prevalence rate of HAV and education status and residency. These results are in agreement with other studies (11  , 19 , 20). The current study has limitation, with regard that the socioeconomic backgrounds of the participants were not mentioned. Furthermore, all hospital employees including physicians, medical and paramedical students, and administrative staffs have not participated in the study. Another limitation that may be referred to is that other hospital’s university-affiliated staffs were not included in this study. In conclusion, approximately 30% of the studied population was susceptible to HAV infection. In view of the changing HAV epidemiology in Iran, the number of unprotected individuals may increase. Thus, periodic screening seropositivity for different populations particularly among hospital staffs is recommended to make the most proper decision for preventative strategies in non-immune persons. Also, in view of increasing in proportion of susceptible young adult and adults and considering that the disease has more severe course as age increases, improvement of standard hygiene and prevention strategies are recommended. Furthermore, vaccination with low cost and cost effective vaccine may play a significant role in the health strategy to protect susceptible healthcare worker populations in the future.
